# Performance Development From Youth to Senior and Age of Peak Performance in Olympic Weightlifting

**DOI:** 10.3389/fphys.2019.01121

**Published:** 2019-08-27

**Authors:** Marianne Huebner, Aris Perperoglou

**Affiliations:** ^1^Department of Statistics and Probability, Michigan State University, East Lansing, MI, United States; ^2^Department of Mathematical Sciences, University of Essex, Colchester, United Kingdom

**Keywords:** weightlifting, performance development, peak performance, age, youth, sex differences, body mass, countries

## Abstract

A total of 3,782 performance results for male and female weightlifters, ages 14–30 from 123 countries, from Youth, Junior, and Senior World Championships and Olympic Games 2013–2017 were used to estimate the age at peak performance in Olympic weightlifting and quantify performance development from adolescence to adulthood. The age at peak performance was estimated for men and women globally and for different geographic regions. Overall, male and female weightlifters achieve their peak performance in weightlifting at similar ages. The median peak age is 26.0 years (95% CI: 24.9, 27.1) for men and 25.0 years (95% CI: 23.9, 27.4) for women, at the 90th percentile of performances. The median peak age was 26.3 years for men (95% CI: 24.5, 29.6) and 26.4 years for women (95% CI: 24.5, 29.6), at the 50th percentile. It is a novel finding that the age at peak performance varies for male and female athletes from different geographic regions (Western Europe, Eastern Europe, Middle East, Far East, North- and South America). For some regions men reach peak performance at a younger age than women, while this relationship is reversed for other regions. A possible explanation could be that socio-economic factors influence the pool of available athletes and thus may under- or overestimate the true peak age. Unlike in track and field where the discipline might determine specific body types, weightlifters at all ages compete in body weight classes, enabling us to compare performance levels and annual rate of change for athletes of different body mass. We quantified increases in performance in Olympic weightlifting for male and female adolescents. Sex-specific differences arise during puberty, boys outperform girls, and there is a rapid increase in their performance levels before the further growth slows down. The largest annual rate of increase in the total weight lifted was achieved between 16 and 17 years of age for both sexes with lower body mass and between 21 and 22 years with higher body mass. Such new information may help to establish progression trajectories for young athletes.

## Introduction

Olympic weightlifting training is comprised of high-speed resistance exercises. It requires technical skills, speed, balance, coordination, and strength. Since muscles exert maximal forces in a fraction of a second, the power output exceeds that of other strength athletes such as bodybuilders and powerlifters (Garhammer, 1993; Storey and Smith, 2012). Performance development in youth depends on changes due to puberty and differ for male and female athletes. Physical fitness and growth during puberty are directly related to hormonal changes, and bone development, muscle strength, and body composition are most effected (Goswami et al., 2014). Participants in Youth World Championships in Olympic weightlifting are 13–17 years old. This is the age range when sex differences in functional capacities for strength, muscular power, and speed become more pronounced (Malina et al., 2010). Despite the highly technical component of Olympic weightlifting the changes in performances are expected to mirror such age and sex related differences. A prior study estimated the age at peak performance from Senior World Championships results (Solberg et al., 2019), but to our knowledge performance curves during the competitive lifespan have not been documented in weightlifting.

Several studies have established performance increases and age at peak performances in track and field disciplines (Hollings et al., 2014; Tønnessen et al., 2015; Boccia et al., 2017; Ganse et al., 2018; Haugen et al., 2018). The performance development varied by discipline and can be attributed to physical growth and learning highly technical skills (Tønnessen et al., 2015). Throwers have higher body mass than other track and field athletes and achieve peak performance at a later age (Hirsch et al., 2016; Haugen et al., 2018). Unlike in track and field, athletes in weightlifting compete in different body weight classes. At all age categories there is one class with no upper limit for the body weight^1^. Thus, athletes can increase their body weight to possibly achieve a higher performance in the lift, although with less efficiency in the body mechanics (Duncan et al., 2013). This enables us to compare performance levels for athletes of different body mass within one sport discipline.

Athletes may discontinue training at competitive levels due to various reasons. Socio-economic factors and availability of public support differ between countries. This impacts athlete development and athlete career termination (Alfermann et al., 2004; Moesch, 2012). In particular, ages of retirement from elite training differ between countries (Kuettel et al., 2017). However, changes in cultural and political landscapes enable athletes to participate, or participate longer, in a sport, in particular women, which has led to a shift in the age of athletes at the Olympic games over time (Elmenshawy et al., 2015). Such factors impact the pool of available athletes and thus may result in differences in peak age for athletes from different geographic and cultural regions.

The aim of this study was twofold. First, we estimated the age at peak performance in Olympic weightlifting for male and female weightlifters globally and in different geographic regions. Since the performance decline in the younger masters age classes (ages 35–45) is similar between men and women (Huebner et al., 2019), we hypothesized that men and women achieve peak performance at the same age and investigated this globally and for different geographic regions. Second, we quantified the age-associated performance development in adolescent athletes stratified by sex, bodyweight, and performance level as measured by percentiles. Due to the differences in peak age in track and field disciplines (Haugen et al., 2018) we hypothesized that athletes with higher body mass would achieve peak performance at a later age.

There are several novel contributions in this study. We quantified the performance growth in Olympic weightlifting for male and female adolescents and established an age range when maximum performance was reached both globally and stratified by geographic regions. We estimated the age at peak performance for athletes of different body mass within one competitive sport discipline for an internationally diverse group. Estimating the rate of performance increase for girls and boys and the age at which the rate of increase is at its maximum stratified by body mass and by performance level may help to establish progression trajectories for youth weightlifters.

## Materials and Methods

### Study Population

**FIGURE 1:**
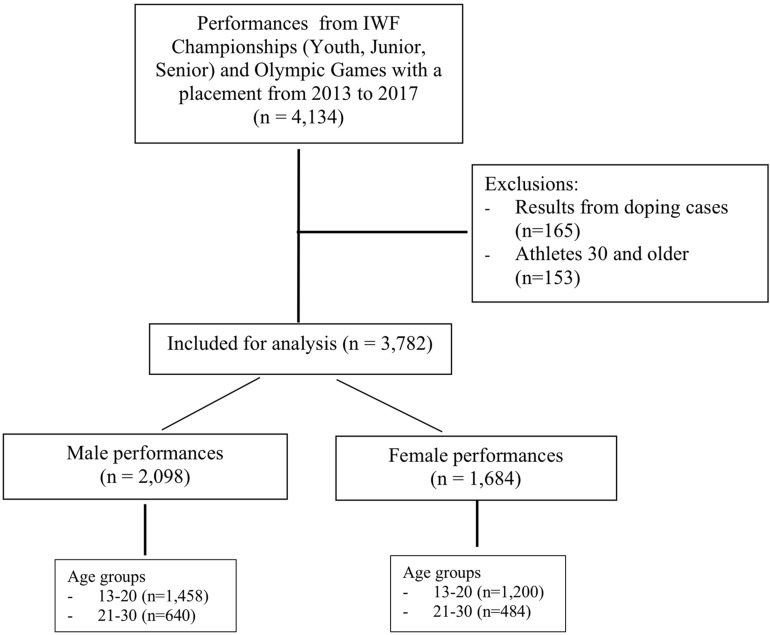
STROBE flow diagram.

Competition results from the International Weightlifting Federation (IWF) Championships and from Olympic Games were included. The competitions are for Youth (ages 13–17), Junior (ages 15–20), and Senior (15 and older) age groups. Data were obtained from the IWF database^2^ from 2013 to 2017. Only a few athletes older than age 30 compete in senior championships, thus leading to sparse matrices for estimation. Therefore, only weightlifters up to age 30 were included in the study. All results from weightlifters who received a sanction due to doping offenses were removed. Sanctioned athletes are listed on the IWF website^2^. Exclusions are described in the study flow diagram (Figure 1).

In competitions the total weight lifted is the sum of the best snatch weight and best clean and jerk weight, if there was at least one valid attempt among three attempts in each of these lifts. The lifts are judged by three referees according to the same rules at all ages. Podium awards are decided within age and body weight categories. Since snatch and clean and jerk results are highly correlated, we analyze the total weight lifted.

The project protocol was reviewed by the Internal Review Board at Michigan State University and was granted an exempt status, since the data are publicly available.

### Statistical Analysis

The performance was defined as the total weight (kg) lifted. Exact body weights were available for each competition result. For comparisons of the age at peak performance overall and in different geographical regions quantile regression models for the performance as a quadratic function of age were fitted with bootstrap sampling to estimate a confidence interval for age at peak performance. There were 500 iterations with random samples of 200 athletes drawn at each iteration. The model for the total weight lifted = a × Age + b × Age^2^ + c used the exact age at the first day of the competition rather than the competition age which is the age as of December 31 or the year of the competition. The maximum was calculated for the 90th and 50th percentiles, and the age at peak performance between ages 19 and 31 was estimated with a 95% bootstrap confidence interval. We are interested in the performance of the top 10% of athletes at the world championships who may have different training ages or different training variables than athletes at lower percentiles. The median (50th percentile) is less likely to be influenced by extreme observations as the mean would be.

Countries with at least 10 results in two of three age categories 16–17, 18–20, and 21–30, or more than 20 results were grouped by geographic regions, so that the combined group was larger than 100. Smaller European countries were included in the West European region. Six regions were considered, Western Europe (Belgium, Denmark, Finland, France, Germany, Ireland, Italy, Netherlands, Norway, Spain, Sweden, United Kingdom), Eastern Europe (Armenia, Azerbaijan, Belarus, Bulgaria, Georgia, Latvia, Poland, Russia, Turkey, Turkmenistan, Ukraine, Uzbekistan), Far East (China, India, Indonesia, Japan, Korea, Malaysia, Mongolia, North Korea, Taiwan, Thailand, Vietnam), Middle East (Egypt, Iran, Iraq, Saudi Arabia, Turkey, Tunesia), North America (Canada, United States), Central/South America (Brazil, Chile, Colombia, Ecuador, Mexico, Peru, Venezuela). Australia and all other countries were labeled as “Other.”

Quantile sheets are an extension of quantile regression models and are an effective tool to estimate curves for median, quartiles, or other quantiles under consideration of other covariates, such as age or body mass. The sheets are based on asymmetrically weighted squares regression but with an extra smoothing dimension to prevent curves at different quantiles from crossing (Schnabel and Eilers, 2013). This enables us to study changes in the conditional distribution of the response variable, namely the total weight lifted, with age as a covariate. Quantile sheets are implemented in R as a special case of a Generalized Additive Model for Location Shape and Scales (GAMLSS) within the R package gamlss (Rigby and Stasinopoulos, 2005). We extended this approach to quantile foliation, to estimate quantiles from 0.05 to 0.95, for two covariates, age and body mass. In order to smooth the data across three dimensions, quantiles, age, and body mass, P-splines are utilized to ensure a smooth function along the axes.

Our findings were reported according to the STROBE statement (von Elm et al., 2007). All analyses were performed using the statistical software R v. 3.5.1 (R Core Team, 2017) and the package gamlss v.5.1.2.

## Results

A total of 3,782 performance results for male and female weightlifters aged 14–30 were used in the analyses (Figure 1). There were 44.6% (*n* = 1,684) female and 55.4% (*n* = 2,098) male weightlifting results 2013–2017 (Table 1). There were 1,085 youth athletes up to age 17 with 51.0% (*n* = 553) males and 49.0% (*n* = 532) females. Athletes from 123 countries participated in the IWF competitions. Most athletes were from East European (23.2%) and Far East countries (24.7%).

**TABLE 1:** Age and body mass distribution stratified by sex in World Championships 2013–2017.

	**Women (*n* = 1684)**	**Men (*n* = 2098)**
**Age groups [*n*, %]**		
13–15	84(5.0%)	40(1.9%)
16–17	448(26.6%)	513(24.5%)
18–20	546(32.4%)	726(34.6%)
21–30	606(36.0%)	819(39.0%)
**Geographic regions [*n*, %]**		
West Europe	133(7.9%)	146(7.0%)
East Europe	378(22.4%)	501(23.9%)
Middle East	117(7.0%)	249(11.9%)
Far East	446(26.5%)	490(23.3%)
North America	121(7.2%)	122(5.8%)
Central/South America	297(17.6%)	288(13.7%)
Other	192(11.4%)	302(14.4%)
**Body mass by age groups (median, range in kg)**		
13–15	51.7(40.6−92.0)	55.3(48.3−83.2)
16–17	57.5(39.9−129.5)	67.9(45.9−151.0)
18–20	60.5(43.4−133.1)	76.2(49.3−166.4)
21–25	62.2(47.1−155.4)	83.3(55.2−171.1)
26–30	62.5(47.3−124.7)	84.4(55.5−173.7)

### Age at Peak Performance in Different Geographic Regions

**FIGURE 2:**
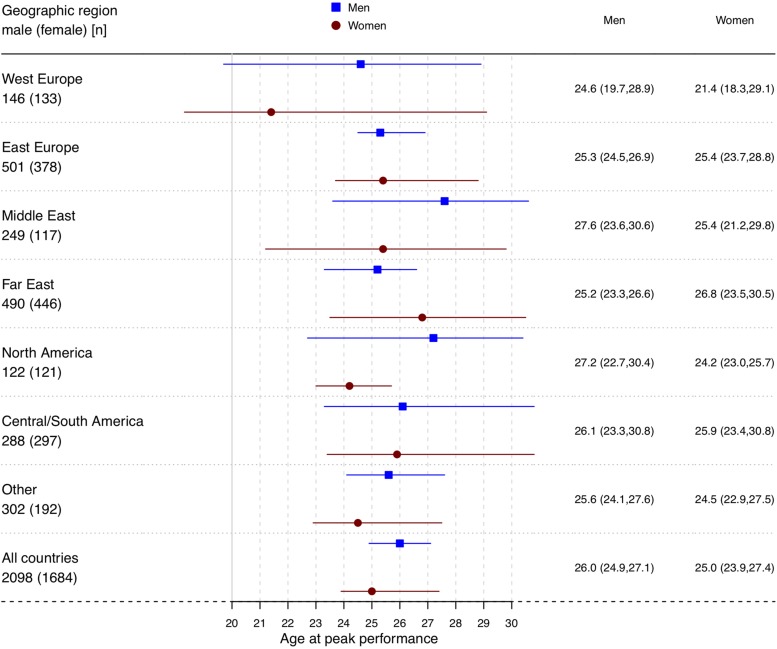
Age at peak performance for 90th percentile performances in different geographical regions. Quantile regression with 95% bootstrap confidence intervals.

From the cross-sectional analysis of world championship results, at the 90th percentile, the age at peak performance was 26.0 years for men (95% CI: 24.9, 27.1) and 25.0 years for women (95% CI: 23.9, 27.4). At the 50th percentile, the peak age was 26.3 years for men (95% CI: 24.5, 29.6) and 26.4 years for women (95% CI: 24.5, 29.6). There was a discrepancy in age of peak performance for different geographical regions. Men from Middle East and North American countries were older with a median of 27.6 and 27.2 years, respectively, while men from Western European countries are younger, 24.6 years, with a wide confidence interval (95% CI: 19.7, 28.9). Women from Western European countries were younger than the average at peak performance with 21.4 years (95% CI: 18.3, 29.1), while women from Far East countries were older with 26.8 years (95% CI: 23.5, 30.5) (Figure 2).

### Rate of Performance Increase by Body Mass and Sex

**FIGURE 3:**
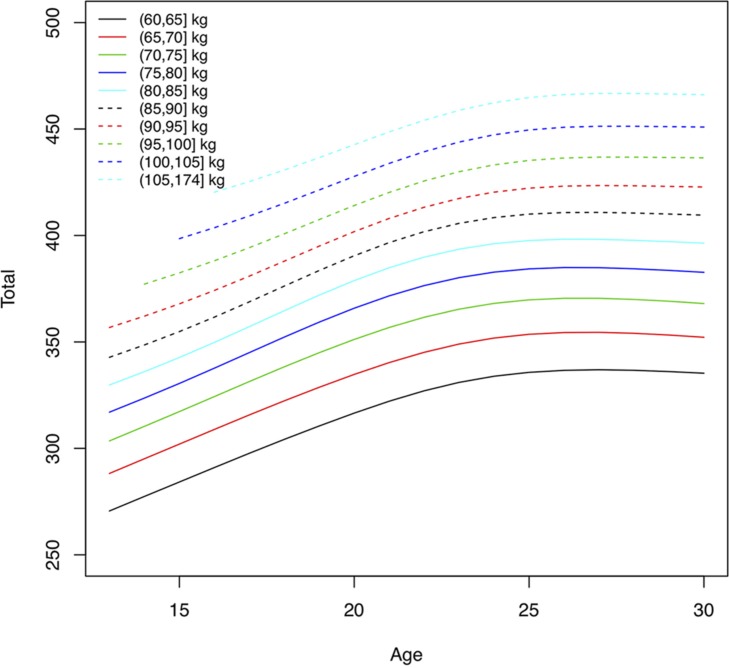
Absolute performance levels in total weight lifted (kg), at the 90th percentile, by age and body mass for men. Lines represent total weight lifted for athletes with different body mass.

**FIGURE 4:**
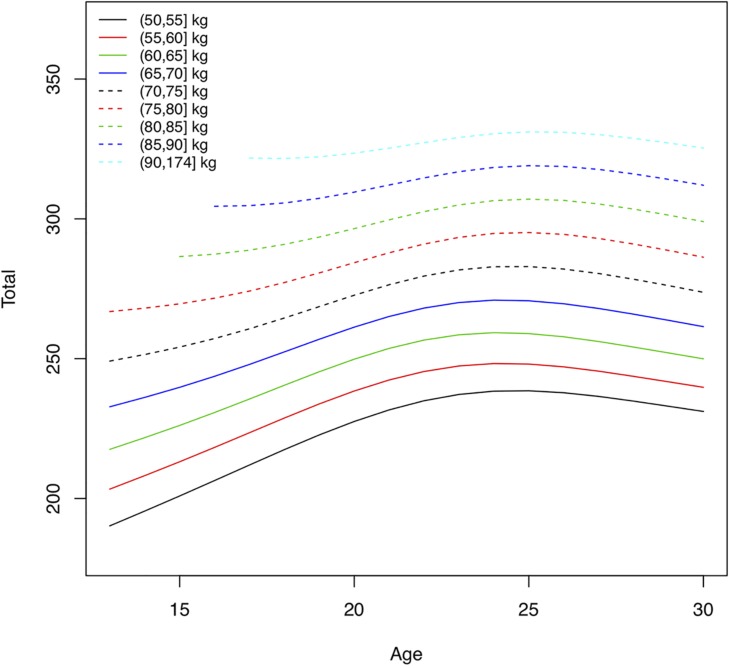
Absolute performance levels in total weight lifted (kg), at the 90th percentile, by age and body mass for women. Lines represent total weight lifted for athletes with different body mass.

**FIGURE 5:**
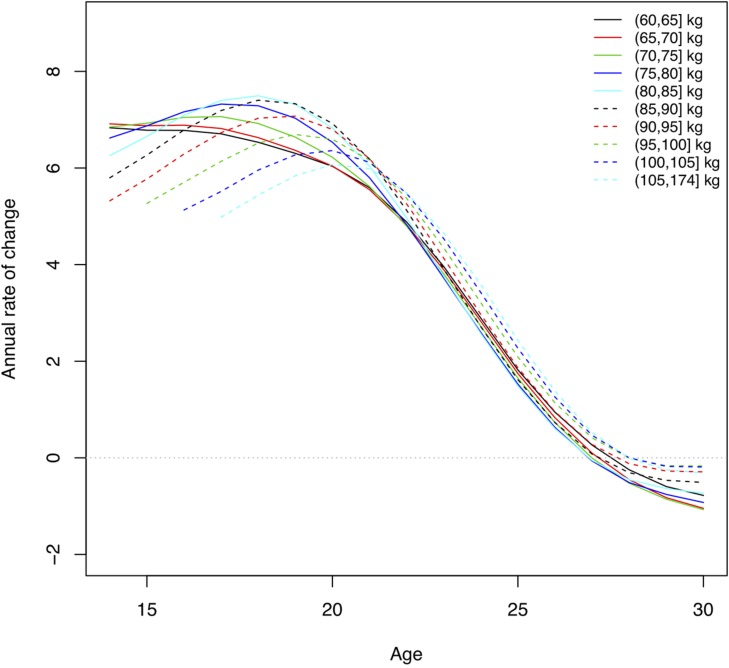
Annual rate of change in performance levels, at the 90th percentile, by age and body mass for men. Lines represent the rate of change in total weight lifted for athletes with different body mass. This corresponds to the derivative of the curves for performance levels, where zero indicates the age at which there was a maximum in the absolute performance level.

**FIGURE 6:**
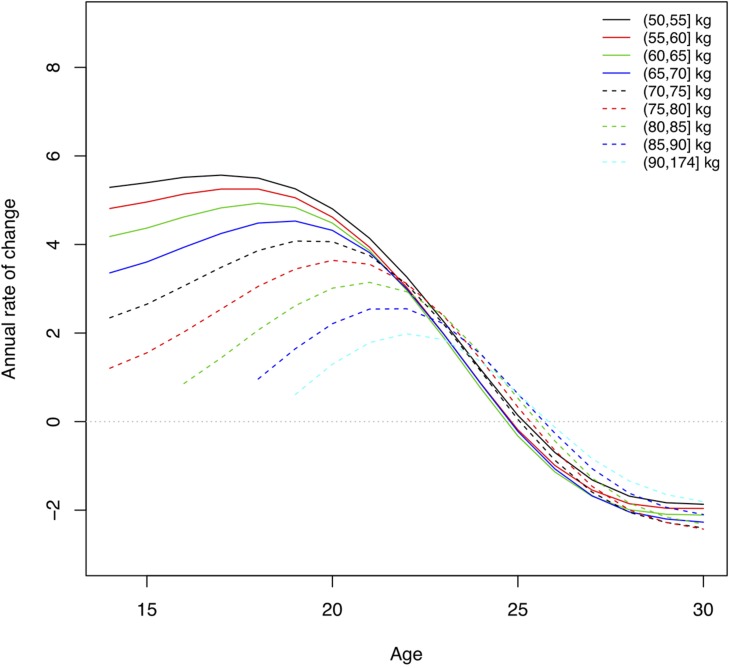
Annual rate of change in performance levels, at 90th percentile, by age and body mass for women. Lines represent the rate of change in total weight lifted for athletes with different body mass. This corresponds to the derivative of the curves for performance levels, where zero indicates the age at which there was a maximum in the absolute performance level.

As they grow older youth weightlifters move into higher body weight classes. The median body weight changed from 49.8 kg at age 14 to 81.5 kg at age 21 for males, and 47.1 kg to 62.0 kg, for females, respectively (Table 1). The total weight lifted increased for male athletes in the middle body mass range (75, 80] kg by 22% from age 16 to peak performance (from 312 to 380 kg total) and for female athletes in the middle body mass range (60, 65] kg by 19% (from 221 to 262 kg total). Using quantile volumes the performance development of adolescent weightlifters can be quantified. Athletes with a higher body mass were able to lift more weight across all ages for both sexes, and performance levels increased with older age (Figures 3, 4). There was a rapid annual rate of increase until ages 16–17 for male and female athletes with lighter body mass (Figures 5, 6 and Table 2). In the unlimited body weight class the age-associated increase was highest at ages 22.1 for females (Figure 5 and Table 2) and 21.3 for males (Figure 6 and Table 2). At age 16 the average annual rate of improvement was 2.2% for boys and 1.6% for girls, respectively. At age 21 the average annual rate of improvement was 1.4 and 1.2% for male and female athletes, respectively.

**TABLE 2:** Performance development, at the 50th and 90th percentiles, for different body weights: age at maximal annual rate of increase and age at peak performance.

	**Women**				**Men**			
	**Age at max increase**		**Age at peak performance**		**Age at max increase**		**Age at peak performance**	
**Body mass intervals (kg)^∗^**	**50th**	**90th**	**50th**	**90th**	**50th**	**90th**	**50th**	**90th**
(50, 55]	14.0	16.8	23.5	24.9				
(55, 60]	15.0	17.2	23.7	24.9	17.0	16.0	24.5	26.6
(60, 65]	16.0	17.6	24.1	24.7	17.4	16.0	24.1	26.6
(65, 70]	17.6	18.5	24.7	24.9	17.8	16.0	24.3	26.6
(70, 75]	19.9	19.5	25.5	25.1	18.2	16.0	24.7	26.6
(75, 80]	21.1	20.5	26.3	25.3	18.9	17.4	25.3	26.2
(80, 85]	21.7	21.1	27.0	25.3	19.9	17.6	26.2	25.7
(85, 90]	21.9	21.7	27.8	25.5	20.7	18.1	27.0	25.5
(90, 95]					21.1	18.7	28.0	25.5
(95, 100]					21.3	19.7	29.0	25.5
(100, 105]					21.3	20.5	29.0	25.5
W (90, 174] M (105, 174]	22.3	22.1	29.0	25.5	21.3	21.1	28.0	25.5

*^∗^Numeric intervals (*a,b*] include all values *x*, so that a < *x* ≤ b.*

### Age at Peak Performance by Performance Level

Female athletes with heavier body mass (>80 kg) reached the peak performance at an earlier age at the 90th percentile (25.5 years) compared to female athletes at the 50th percentile (27.8 years). The peak ages were comparable for the 90th and 50th percentile in the lighter body mass groups (24–29 years). Male athletes at the 50th percentile reached the peak performance at an older age (24–29 years) compared to those at the 90th percentile (25–26 years). Female athletes at the 50th percentile reached the peak performance at an older age (24–29 years) compared to those at the 90th percentile (24–25 years). Women and men reached the age at peak performance at similar ages. In the unlimited body weight category men and women were 25.5 years at peak performance (Table 2). At the 50th percentile male athletes with higher body mass reached their peak performance at a later age than athletes with a lower body mass, but ages were more similar for weightlifters with different body masses at the 90th percentile.

## Discussion

We described performance development in competitive athletes in Olympic weightlifting from adolescence to career peak. We focused on analyzing performances in recent years, 2013–2017, from World Championships and Olympic Games since there has been an influx of youth and women into the sport of weightlifting, and improvements in performances were seen over time (Elmenshawy et al., 2015; Huebner et al., 2019). A total of 3,782 performances were analyzed with athletes from 123 countries (females 42–44%).

### Age at Peak Performance

Performance decline in weightlifting after age 30 has been studied (Meltzer, 1994; Huebner et al., 2019). It has been shown that women’s decline in performance mirrors that of men, except for an accelerated decline from late 40’s to late 50’s coinciding with a transition into menopause. This raises the question whether men and women achieve peak performance prior to age 30 at the same age. In our study the age at peak performance, at the 90th percentile, was similar, with overlapping confidence intervals, for men and women, namely 26.0 years for men (95% CI: 24.9, 27.1) and 25.0 years for women (95% CI: 23.9, 27.4), respectively. The corresponding peak age for the 50th percentile of performances was 26.3 years for men (95% CI: 24.5, 29.6) and 26.4 years for women (95% CI: 24.5, 29.6). This is comparable to the mean age at peak performance (±90% confidence limits) of 26 ± 3 years which was estimated using Senior World Weightlifting Championships data from 1998 to 2017 (Solberg et al., 2019). In comparison, in track and field disciplines the mean age at peak performance (±90% confidence limits) was also similar for men and women in sprint and hurdles with 25.2 ± 0.3 and 25.7 ± 0.3 years and in jumping disciplines with 25.8 ± 0.3 and 25.6 ± 0.4 years, respectively. In throwing disciplines men reached their peak at an older age, 28.0 ± 0.4 years, both in comparison to women with 26.7 ± 0.6 years, and in comparison to athletes from other disciplines (Hollings et al., 2014). Thus the overall age at peak performance in weightlifting was comparable to that of sprint and jumping disciplines in track and field.

### Geographic Differences for Male and Female Athletes

The estimated median age at peak performance varied for athletes from different countries. This is a new finding about weightlifters from an international database. The oldest age at peak performance was 27.6 years for men in the Middle East, 27.2 years for men in North America, and 26.8 for women in the Far East. West European women were younger at peak performance than East European women with 21.4 and 25.4 years, respectively. This corresponds to observed younger age at peak performance for German and Italian track and field athletes. German athletes reached the maximal performance at an average age of 20.0 years for men and 21.6 years for women (Ganse et al., 2018), while Italian top-level high jumpers were 21.6 and 21.1 years for men and women, respectively (Boccia et al., 2017). It is unlikely that such differences between geographic regions are solely due to biological reasons or training variables. Participation requires financial resources for travel and fees, and many countries have favored boys in organized sports (Tremblay et al., 2014). Athlete development and career termination is impacted by public support, cultural expectations, and life transitions (Alfermann et al., 2004; Moesch, 2012; Kuettel et al., 2017). Women are more likely to discontinue than men because of family related reasons (Moesch, 2012). Such factors change over time, therefore we used recent data from 2013 to 2017. In North America and Western Europe women were younger than men at peak performance with 24.2 years and 27.2 years, respectively, in North America and 21.4 and 24.6 years for women and men, respectively, in West European countries. However, women were older than men in Far East countries with 26.8 years for women compared to 25.2 years for men. Using all countries to estimate the age at peak performance results in overlapping confidence intervals for men, 24.9–27.2 years, and women, 23.9–27.1 years. The observed differences in peak age may be due to participation levels of women in different countries. Although confidence intervals for peak ages in the geographic regions overlap, the width of the confidence intervals and the estimated median peak ages differ for different regions. This may be an indicator that the global median age at peak performance may be an under- or overestimate, if the pool of active athletes does not reflect the physical capabilities of the population due to participation levels. This may be true for estimated age at peak performance in other sport disciplines that did not take geographic differences into account.

### Rate of Performance Increase by Body Mass and Sex

Performance levels improve rapidly from adolescence to adulthood. In this study the total weight lifted increased for male athletes in the middle body mass range (75, 80] kg by 22% from age 16 to peak performance and for female athletes in the middle body mass range (60, 65] kg by 19%. At age 16 the average annual rate of improvement across body weight groups was 2.2% for boys and 1.6% for girls, at the 90th percentile. At age 21 the average annual rate of improvement was 1.4 and 1.2% for male and female athletes, respectively. A higher annual rate of improvement for boys compared to girls is not surprising, since the total performance increases dramatically in puberty for boys compared to girls (Figures 3, 4). However, at the 50th percentile, the average annual improvement was 2.4% for boys and 3.0% for girls in our study, which is comparable to the rates observed for snatch and clean and jerk based on (arithmetic) mean performance (Solberg et al., 2019). This highlights the differences we have seen in our study (Table 2) between top athletes, e.g., 90th percentile compared to athletes competing at a high level at world championships but not at the top. Our analyses also distinguished results for athletes with different body mass. The largest annual rate of increase in the total weight lifted was achieved between 16 and 17 years of age for both sexes with lower body mass. The age-associated increase was highest at ages 21–22 for athletes with higher body mass. After reaching the maximum annual change the performance levels continued to increase but in smaller increments each successive year until the peak performance was reached.

### Performance Development and Body Mass and Performance Level

The mean body mass for 18–30-year-old weightlifters at the IWF World Championships was 83.4 ± 23.9 kg for men (median 76.8) and 65.2 ± 17.6 kg (median 62.0) for women. In comparison, collegiate track and field athletes had a mean body mass of 78.4 ± 11.6 kg for men and 67.0 ± 14.2 kg for women. Throwers had the highest body mass of for men and women overall 90.4 ± 18.3 kg (Hirsch et al., 2016). Thus, female weightlifters and female track and field athletes had similar average body mass, and male weightlifters were lighter than throwers. In track and field disciplines physique varies by discipline and higher body mass is critical for success in throwers (O’Connor et al., 2007; Hirsch et al., 2016). Athletes in Olympic weightlifting compete in different body weight classes and thus can be competitive within a range of morphological characteristics. The performance development curves showed that weightlifters with higher body mass were able to lift more weight across ages. The maximum annual rate of performance increase was reached at ages 15–22, younger for athletes with lower body mass and older for athletes with higher body mass. This was similar for athletes at the 50th and the 90th percentile. Top-level male and female weightlifters, in the 90th percentile, with higher body mass reached their peak performance at a younger age (25 years) than weightlifters with higher body mass in the 50th percentile (28–29 years). An older age of peak performance (30–31 years) was observed in second tier, and thus lower percentile, German weightlifters (Faber, 2012). Differences in ages of peak performance for different body weight classes was also observed for snatch and clean and jerk (Solberg et al., 2019).

### Limitations

There are limitations to our study. First, there may be a selection bias due to socio-economic factors leading adolescent and young adult weightlifters to discontinue training due to experiencing life events, such as transition to university, employment status, changes in personal relationships, or financial reasons. This could lead to an estimated age at peak performance that is younger than it would be if athletes were able to continue their careers until they reach their full potential. However, using an internationally diverse group as in our study may help balance cultural effects. Second, the athlete selection to participate in international competitions depend on numerous factors, and countries may handle this differently. While there could be higher performances at other competitions, the judging of performances at world championships is under uniform conditions that cannot be guaranteed in other events or circumstances. Third, anthropometric and training variables were not considered. Thus, body mass is the only indicator of the physique of the athlete. However, this enabled us to compare performance levels for athletes of different body mass which has not been distinguished within track and field disciplines to the best of our knowledge. Fourth, while undetected doping violations cannot be excluded from the data in our study, we excluded all results from athletes who were found to violate anti-doping policies at any one time point.

## Conclusion

We quantified performance development in Olympic weightlifting for male and female adolescents and established an age range when maximum performance is reached. The age at peak performance differed for athletes from various geographical regions. Women and men reach their peak weightlifting performance at similar ages. Performance development differs for athletes with different body mass. The annual rate of performance increase was at its maximum in the mid-teens for athletes with lower body mass and in their early 1920s for athletes with higher body mass. This study provides new information related to age-associated performance increase for youth weightlifters stratified by body mass and sex. Such results may help to establish progression trajectories for young athletes. Further research is warranted to examine gender differences and socio-economic factors as they relate to age at peak performance.

## Data Availability

The datasets for world championships can be accessed from the website of the International Weightlifting Federation (https://www.iwf.net/new_bw/results_by_events/). Athletes with sanctions are listed for international athletes (https://www.iwf.net/anti-doping/sanctions/).

## Ethics Statement

The studies involving human participants were reviewed and approved by the IRB, Michigan State University. Written informed consent from the participants’ legal guardian/next of kin was not required to participate in this study in accordance with the national legislation and the institutional requirements.

## Author Contributions

MH contributed to the concept. MH and AP performed the data analysis, interpreted the results, and wrote and approved the manuscript.

## Conflict of Interest Statement

The authors declare that the research was conducted in the absence of any commercial or financial relationships that could be construed as a potential conflict of interest.

## Footnotes

1 https://www.teamusa.org/usa-weightlifting/weightlifting101/weight-classes

2 http://www.iwf.net/
